# Anti-inflammatory and cancer chemopreventive potential of essential oils from some cultivated plants in Egypt

**DOI:** 10.1038/s41598-026-35195-0

**Published:** 2026-01-29

**Authors:** Mohammed I. Ali, Ahmed R. Hamed, Emad M. Hassan, Faten M. Abou elella, Sayed A. El-Toumy, Ahmed M. Aboul-Enein

**Affiliations:** 1 Medicinal and Aromatic Plants Research Department, National Research Centre, 33 El- Bohouth St., Dokki, Giza, 12622 Egypt; 2 Chemistry of Medicinal Plants Department, National Research Centre, 33 El-Bohouth St., Dokki, Giza, 12622 Egypt; 3 Biochemistry Department, Faculty of Agriculture, Cairo University, Giza, 12613 Egypt; 4 Chemistry of Tannins Department, National Research Centre, 33 El-Bohouth St., Dokki, Giza, 12622 Egypt

**Keywords:** Essential oils, Medicinal plants, Anti-inflammatory and cancer chemopreventive, Cancer, Drug discovery, Plant sciences

## Abstract

This study aimed to investigate the biological activity of essential oils (EOs) of *Artemisia abrotanum*, *Lavandula dentata*, *Cymbopogon citratus* and *Laurus nobilis* as anti-inflammatory and cancer chemopreventive activities. The anti-inflammatory activity was evaluated using lipopolysaccharides-induced nitric oxide (NO) inhibition on murine macrophage cells (RAW264.7) and cancer chemo-preventive influence was assessed in vitro utilizing Hepa1c1c7 murine carcinoma cells. The EO of *A. abrotanum* has the most potential activity toward inhibition of NO release, recording 96.6 ± 0.1%, as estimated by Greiss assay. Followed by EOs of *L. dentata* and *L. nobilis* with 63.6 ± 0.11 and 37.0 ± 0.23%, respectively. At the protein expression level, western blotting technique was used to evaluate the expression of iNOS. The EO of *A. abrotanum* at 100 µg/ml exhibited a very high impact on inhibiting iNOS expression, followed by EOs of *L. dentata* and *L. nobilis*. On the other hand, pre-screening concentration (100 µg/ml) revealed that the EOs of *L. dentata* and *A. abrotanum* have moderate potency to induce expression of chemo-preventive marker NQO1. The results revealed that the EO of *A. abrotanum* had strong anti-inflammatory activity. While the EOs of *L. dentata* and *A. abrotanum* have moderate potency to induce cancer chemoprevention.

**Supplementary Information:**

The online version contains supplementary material available at 10.1038/s41598-026-35195-0.

## Introduction

Inflammation is a natural, complicated and vital process for tissue defense and repair. It is a crucial innate immune response that safeguards the body against infection and injury by activating immune cells and molecular signals to the injured tissues. Acute inflammation has a preventive and self-limiting function. However chronic inflammation can be injurious and is linked to numerous disorders including cancer^1^. The inflammatory process results from cellular and vascular events induced by substances known as inflammatory mediators. They are vital molecules that coordinate the intricate processes of inflammation, balancing defense and tissue healing. Among these molecules are nitric oxide (NO), cytokines, chemokines, eicosanoids and vasoactive amines^2^.

Persistent activation of inflammatory pathways has been recognized as a critical driver in the multistep process of carcinogenesis. Plants are thus increasingly recognized for their potential in chemoprevention due to active phytochemicals that can simultaneously enhance cellular defense mechanisms and attenuate inflammation. Many studies have explained in detail the strong link between chronic inflammation and cancer development^3–6^. Chronic injury due to inflammation also causes alteration of signal transduction proteins, disruption of DNA integrity and the possible activation of pathways with carcinogenic potential^4^. Inflammation confers on the microenvironment a cancer-friendly atmosphere through activating various pro-inflammatory signaling molecules, and targeting inflammatory signaling thus remains one of the promising approaches for cancer prevention^6^.

Cancer chemoprevention involves the application of diverse modalities, both natural and synthetic, to inhibit, prevent or postpone cancer by obstructing tumor formation in its early stages or by decelerating its growth rate, thus deferring the malignant properties and behavior of tumors. This prevention includes interrupting or delaying multiple molecular pathways, among which is the nuclear factor E2-related factor 2 (Nrf2) pathway. When Nrf2 is activated in the Keap1/Nrf2 signaling pathway, it translocates to the nucleus and attaches to the antioxidant response elements (ARE) of target genes. Nrf2 regulates the expression of several enzymes associated with phase II metabolism and safeguards cells against oxidative damage. These enzymes include heme oxygenase 1 (HO-1), NADPH: quinone oxidoreductase 1 (NQO1) and γ-glutamylcysteine synthetase (γGCS). NQO1 generates reduced forms of tocopherols and ubiquinone, making them effective in neutralizing free radicals. HO-1 serves a crucial antioxidant function in preserving cellular homeostasis against ROS^7,8^.

There are a number of plant-based phytochemicals known to activate the transcription factor Nrf2, which controls the expression of NQO1. The expression of detoxification enzymes in response to pro-carcinogens is mediated through the Nrf2/Keap1 signaling pathway, considered one of the most important mechanisms by which the cell defends against carcinogenic stress^9,10^.

Natural products derived from bacteria, marine organisms and plants have been essential for the development of inflammation and cancer treatments due to their wide chemical diversity, unique structural characteristics and biological efficacy, which exhibit less toxicity. These products become great importance in the prevention and treatment of cancer, which induce apoptosis, inhibit cell invasion and metastasis, exhibit anti-inflammatory properties and enhance immune responses^11^.

Medicinal and aromatic plants have played a vital role in the healthcare practices of various civilizations throughout human history, providing natural treatments and serving as a fundamental resource for the development of current pharmaceuticals^12^. In the last few years, much attention has been given to the essential oils (EOs) and pharmacological activities of aromatic plants. Many studies suggest that the pharmacological activities of these plants are particularly related to the chemical composition of their EOs. Essential oils exhibit significant therapeutic potential, especially in cancer therapy. These oils have bioactive substances, chiefly terpenoids and phenylpropanoids, which demonstrate antioxidant, anti-inflammatory and antimicrobial capabilities, providing defense against several metabolic and infectious disorders^13,14^.

Among the promising species were *Artemisia abrotanum* L. “Asteraceae, commonly known as southernwood”, *Lavandula dentata* L. “Lamiaceae, commonly known as lavender”, *Cymbopogon citratus* (DC.) Stapf “Poaceae, commonly known as lemongrass” and *Laurus nobilis* L. “Lauraceae, commonly known as bay laurel”.

Previous studies on the extracts prepared from these species: “*A. abrotanum*, *L. dentata*, *C. citratus* and *L. nobilis*” demonstrated various biological activities including analgesic, antioxidant, anti-inflammatory, anti-proliferative, antidepressant, anti-diabetic, anti-asthma, hypolipidemic, hypoglycemic, anti-acetyl-cholinesterase, anti-nociceptive, antidiarrheal, antimalarial, immunosuppressant effect, apoptotic effect, antimicrobial and antiviral activities^15–34^. Furthermore, EOs distillated from these species showed different biological activities such as antioxidant, cytotoxic, antimicrobial, anticoagulant, analgesic and anti-diabetic, hypotension, vasorelaxation, anxiolytic, anticonvulsant, depressant, neuroprotective, anti-inflammatory, anticholinesterase activities and reduced the blood cholesterol level^35–53^.

According to our previous publication on the chemical composition of these essential oils^54^, the most prominent constituents of *C. citratus* were *E*-citral (36.7%) and *Z*-citral (32.2%), followed by *β*-myrcene (8.8%). Whereas, the major constituents of *L. dentata* were eucalyptol (31.2%), camphor (19.7%) and *α*-pinene (5.5%). On the other hand, the most prominent compounds of *L. nobilis* were eucalyptol (35.5%), *α*-terpinyl acetate (16.3%) and sabinene (10.6%). Also, *A. abrotanum* has artemisia ketone (31.8%), *α*-eudesmol (20.6%) and *γ*-eudesmol (5.1%) as the most prominent constituents.

In the present study, we investigated the biological efficacy of the essential oils of southernwood, lavender, lemongrass and bay laurel, with a focus on their anti-inflammatory activity and potential roles in cancer chemoprevention. A robust cell-based model of lipopolysaccharide (LPS)-induced inflammation in RAW264.7 macrophages was employed to assess anti-inflammatory activity. While chemopreventive potential was evaluated by measuring the induction of NQO1, a principal marker of Nrf2 activation, in the murine Hepa1c1c7 cell line.

## Experimental

### Chemicals and reagents

Dulbecco’s modified Eagle’s medium (DMEM), *α*-modified minimum essential medium Eagle (*α*-MEM), Trypsin-Versene (EDTA) Mix, Dulbecco’s Phosphate-Buffered Saline (DPBS), L-Glutamine and penicillin/streptomycin stock (Lonza Verviers SPRL, Belgium). Fetal Bovine Serum (FBS; SeraLab, UK); Lipopolysaccharide (LPS; Sigma-Aldrich, from *Escherichia coli* serotype O111:B4), Enzyme chemiluminescence (ECL; Thermofisher scientific, USA) and Tris-buffered saline with 0.1% Tween 20 (TBST).

### Plant materials

The aerial parts of *L. dentata*, *A. abrotanum* and *L. nobilis* were collected from the experimental farm of the National Research Centre in December, while *C. citratus* was collected from Agricultural Research Station in Al-Qanatir Al-Khairiya in December. Plants kindly identified by Dr. Mohamed El-Gebally, former Researcher of Botany, National Research Centre, Dokki, Cairo, Egypt. The specimens are stored in the herbarium of National Research Centre under the voucher numbers; M277 for *A. abrotanum*, M278 for *C. citratus*, M279 for *L. nobilis* and M280 for *L. dentata*.

### Extraction of EOs

One hundred grams of fresh aerial parts of collected plants were subjected to water distillation using Clevenger’s apparatus. The distillation persisted for 3 h from the onset of boiling. The volatile substances were collected, dehydrated using traces of anhydrous sodium sulfate, filtered and preserved at -20℃ until required^55^.

### Cell culture

Murine macrophage (RAW264.7) and murine hepatoma (Hepa1c1c7) cells were purchased from ATCC^®^. RAW264.7 cells were cultured as monolayer in DMEM medium enriched with 10% (v/v) heat-inactivated FBS. Hepa1c1c7 cells were cultured as a monolayer in *α*-MEM medium enriched with 10% (v/v) heat and charcoal–inactivated FBS. Both media supplemented with 100 µg/ml streptomycin sulfate, 100 U/ml penicillin and 4 mM L-glutamine. The cells were incubated in a humidified CO_2_ incubator with 5% CO_2_ at 37℃.

### Anti-inflammatory activity

A model for the inhibition of LPS-induced NO release in RAW264.7 was carried out as previously described^56,57^. Briefly, cells (1.5 × 10^6^ cells/well) were plated in 6-well plates and incubated for 24 h. On the following day, cells were treated with either 0.1% v/v DMSO (negative control, LPS^−^), 100 ng/ml LPS (LPS^+^) or LPS combined with samples (100 µg/ml), then incubation for 24 h. Indomethacin (250 µM) served as the positive control.

#### iNOS activity assay

The Griess assay^58^ was utilized to determine NO in culture supernatants after 24 h. Briefly, 100 µl of culture supernatant from each well were combined with an equal volume of Griess reagent, mixed at room temperature and absorbance was measured at 520 nm using a TriStar2 LB 942 microplate reader (Berthold Technologies, Bad Wildbad, Germany). The percentage of inhibition was calculated relative to the LPS only group (LPS^+^). The following formula was used to calculate the % inhibition of NO release:

$$\text{Inhibition of LPS} - \text{induced NO}\ (\%) = \frac{{\rm Ab} - {\rm As}}{{\rm Ab}} \times 100$$

Whereas; Ab: Absorbance of LPS^+^. As: Absorbance of sample.

#### Preparation of cell lysate for the assessment of iNOS protein expression

Western blotting analysis was employed to assess the protein expression of the pro-inflammatory marker, inducible nitric oxide synthase (iNOS). After exposure for 24 h as mentioned above, cells were rinsed with ice-cold DPBS. Cells were subsequently scrapped in ice-cold homogenization buffer (25 mM Tris-Cl, pH 7.4, 250 mM sucrose) supplemented with protease/phosphatase inhibitor cocktail (Halt^®^, Thermo-fisher scientific, USA) and transported to appropriately labeled micro-centrifuge tubes. Cell suspensions were subsequently sonicated on ice for 5 s at 30% amplitude. Cell lysates were then centrifuged at 12,000 ×g for 5 min and the supernatants (cytosolic fractions) were collected. Total protein content was assessed using a NanoDrop spectrophotometer (Thermo Fisher Scientific Inc., Waltham, Massachusetts, USA). Samples were then stored at -80℃ freezer until assayed for protein expression utilizing western blotting technique.

### Cancer chemopreventive activity

The induction of NAD(P)H: quinone oxidoreductase 1 (NQO1) and heme oxygenase 1 (HO-1) in Hepa1c1c7 cells was assessed^59^. Briefly, cells (3 × 10^5^ cells/ml) were plated in 6-well plates and incubated overnight to adhere and form semi-confluent monolayers. Monolayers were treated with either 0.1% v/v DMSO (negative control), samples or 4‵-bromoflavone (4‵-BF, 10 µM) served as positive control for 4 h (HO-1) or 48 h (NQO1). Subsequently, treatment media were discarded and monolayers were rinsed with ice-cold DPBS (2 ml/well). Cells were then scrapped in ice-cold lysis buffer (5 µM FAD, 250 mM sucrose and 25 mM Tris-Cl, pH 7.4) and transported to appropriately labeled micro-centrifuge tubes. Cell suspensions were subsequently sonicated on ice for 5 s at 30% amplitude. Cell lysates were then centrifuged at 12,000 ×g for 5 min and the supernatants (cytosolic fractions) were collected. Total protein content was assessed using a NanoDrop spectrophotometer (Thermo Fisher Scientific Inc., Waltham, Massachusetts, USA). Samples were then stored at -80℃ freezer until assayed for protein expression utilizing western blotting technique.

### Western blotting technique

Proteins were resolved under denaturing conditions by sodium dodecyl-sulfate polyacrylamide gel electrophoresis (SDS-PAGE) on a 10% acrylamide/bisacrylamide gel and electrophoresed for 90 min at 110 volts using a Biorad Tetra Cell (Biorad, USA). Subsequently, the resolved proteins were transferred to nitrocellulose membrane (0.45 μm pore size) using a Biorad transfer module at 100 volts for 60 min. Following electro-blotting, blots were cut alongside the Mwt. ladder onto appropriate strip for each protein target. Membrane strips were then blocked for 1 h with 5% non-fat milk. Strips were then incubated at 4℃/overnight with corresponding primary antibodies against iNOS (1:1000, Biomatik, Canada), NQO1 (1:1000, Elabscience, USA), HO-1 (1:500, Thermo-fisher scientific, USA) or *β*-actin (1:2000, Thermo-fisher scientific, USA). Then, 3 × 5 min washes with TBST, the membranes were incubated with appropriate horseradish peroxidase-conjugated secondary antibodies for 1 h at room temperature, followed by 3 × 5 min washes with TBST. Membrane proteins were developed utilized ECL western blotting substrate for 5 min, subsequently imaging was conducted utilizing a UVP Biospectrum Imager (Analytik Jena, Germany).

A dose-response experiment was performed on samples demonstrated high protein expression utilizing concentrations of 6.25, 25 and 100 µg/ml.

### Statistical analysis

Statistical analysis of the data was conducted utilizing one-way analysis of variance (ANOVA) and Dunnett’s multiple comparisons test *via* GraphPad Prism^®^ V6.0 software (GraphPad Inc., San Diego, USA). The results are expressed as mean ± standard error of the mean (SME). The statistical significance was determined at *P* < 0.05.

## Results

### Evaluation of anti-inflammatory potential

The innate immune system can be activated by lipopolysaccharide (LPS), which is known to cause inflammation^60^. In macrophages, LPS interacts with the Toll-like receptor 4 (TLR4), which leads to the synthesis of many pro-inflammatory mediators, involving nitric oxide (NO) which is released by the inducible nitric oxide synthase (iNOS) enzyme^61^.

In this study, we used the murine macrophage RAW264.7 model as a robust and inducible cell for the induction of inflammation by LPS, which is a type recognition molecule and a component of the cell wall of gram-negative bacteria^62^.

#### iNOS activity assay (Greiss assay)

**Fig. 1:**
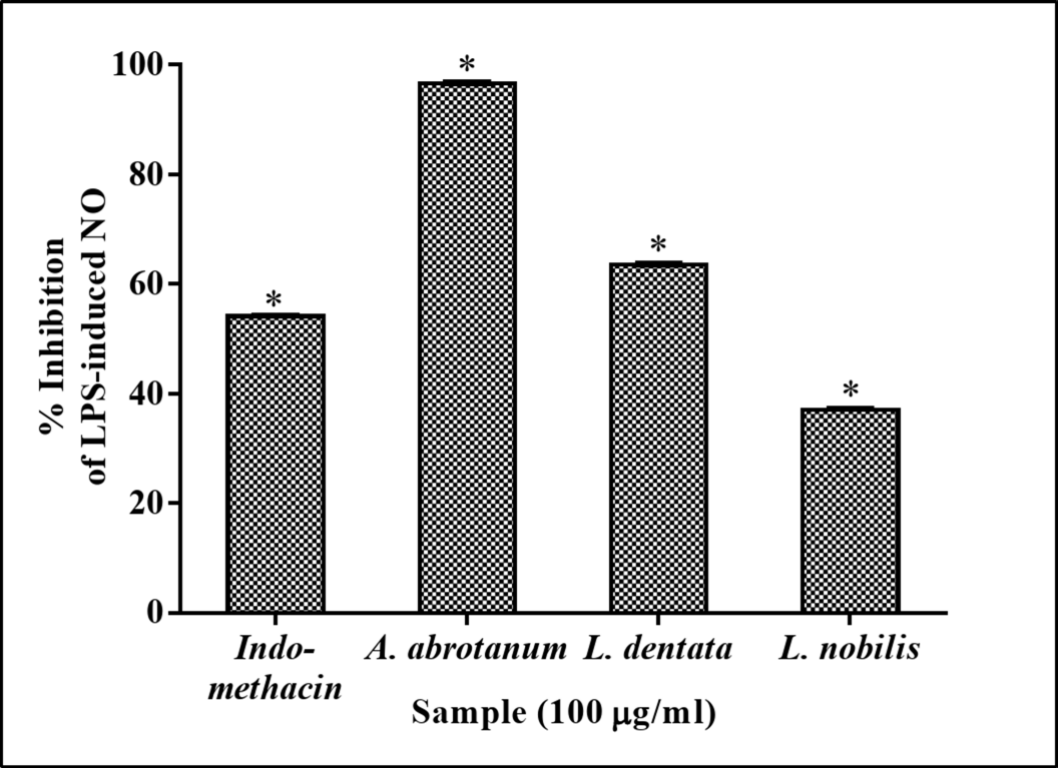
Inhibition of LPS-induced NO in RAW264.7 cells. Indicated cells were cultured as monolayers and treated as mentioned in the Materials and Methods section. Data are means ± SEM (*n* = 2). * denotes significance level of *P* < 0.05 (one-way ANOVA and Dunnett’s test).

The EOs were initially pre-screened in vitro at 100 µg/ml for their anti-inflammatory potential. As displayed in Fig. 1, the pre-screening revealed that the EO of *A. abrotanum* has the most potential activity for inhibition of NO release, recording 96.6 ± 0.1% inhibition of LPS-induced NO, as estimated by the Greiss assay. Whereas, both EOs of *L. dentata* and *L. nobilis* exhibited 63.6 ± 0.11 and 37.0 ± 0.23% inhibition of LPS-induced NO, respectively. Furthermore, the EO of *C. citratus* exhibited a strong cytotoxic effect against RAW264.7 cells.

In addition, indomethacin (250µM) was used as a positive drug and showed 54.1 ± 0.11% inhibition of LPS-induced NO.

#### Protein expression level

**Fig. 2:**
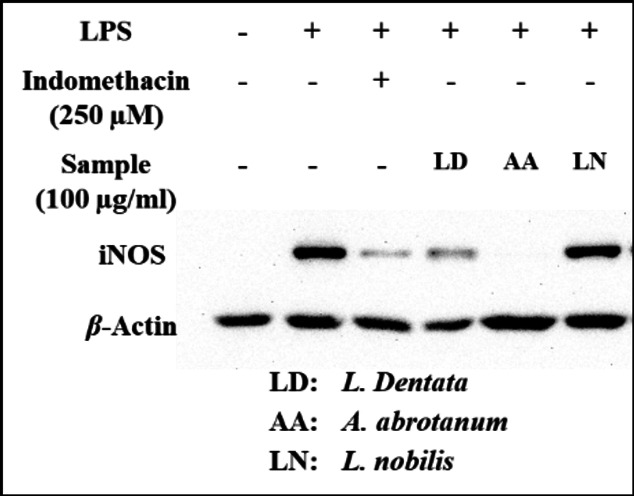
Effect of essential oils (100 µg/ml) on LPS-induced protein expression of iNOS in RAW264.7 cells. Indicated cells were cultured as monolayers and treated as mentioned in the Materials and Methods section. An uncropped image of all blots shown is provided in supplementary material as Figure S1.

At the protein expression level, a western blotting technique was used to confirm the NO inhibition at the level of iNOS protein expression. The EO of *A. abrotanum* at 100 µg/ml exhibited a very high impact on the inhibition of iNOS expression, followed by EOs of *L. dentata* and *L. nobilis* as shown in Fig. 2.

**Fig. 3:**
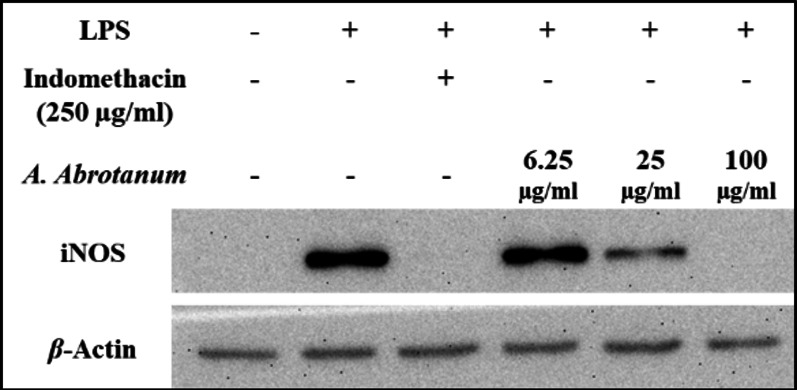
Effect of EO of *A. abrotanum* in gradual concentration on LPS-induced protein expression of iNOS in RAW264.7 cells. Indicated cells were cultured as monolayers and treated as mentioned in the Materials and Methods section. An uncropped image of all blots shown is provided in supplementary material as Figure S2.

An additional experiment was performed on the EO of *A. Abrotanum*, which showed the greatest ability to inhibit iNOS protein expression, with increasing concentrations (6.25, 25 and 100 µg/ml) and showed concentration-dependent inhibition of iNOS protein expression induced by LPS, as displayed in Fig. 3. The highest inhibitory effect of EO of *A. Abrotanum* at 100 µg/ml, followed by 25 and 6.25 µg/ml on iNOS protein expression.

### Evaluation of cancer chemoprevention

NAD(P)H: quinone oxidoreductase 1 (NQO1) is an antioxidant enzyme that plays an important role in cancer chemoprevention. NQO1 preserves cells from oxidative stress by converting quinones into hydroquinones^63^. The nuclear factor‑erythroid factor 2‑related factor 2 (Nrf2) stimulates NQO1, heme oxygenase 1 (HO-1) expressions and other cytoprotective enzymes in reacting to cellular stress^8^.

**Fig. 4:**
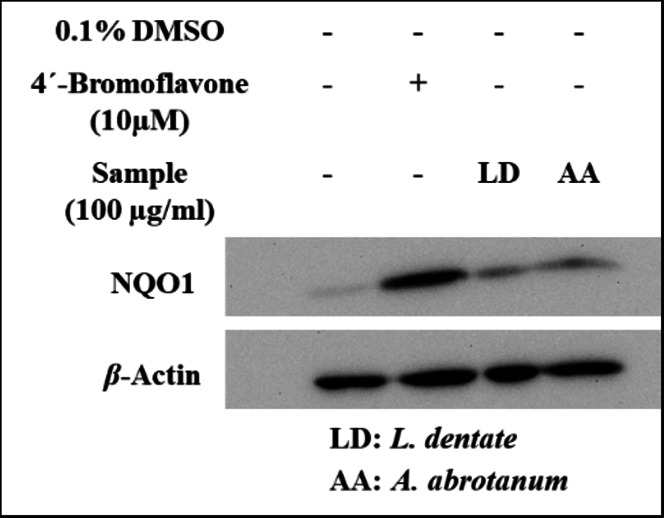
Up-regulation of NQO1 protein expression by essential oils (100 µg/ml) in Hepa1c1c7 cells. Indicated cells were cultured as monolayers and treated as mentioned in the Materials and Methods section. An uncropped image of all blots shown is provided in supplementary material as Figure S3.

Treatment of Hepa1c1c7 cells with a pre-screening concentration (100 µg/ml) revealed that the EOs of *L. dentata* and *A. abrotanum* have moderate potency to induce expression of chemo-preventive marker NQO1 as evaluated by western blotting technique, as illustrated in Fig. 4.

Whereas, the EOs of *C. citratus* and *L. nobilis* exhibited a strong cytotoxic effect against Hepa1c1c7 cells as observed under an inverted microscope.

**Fig. 5:**
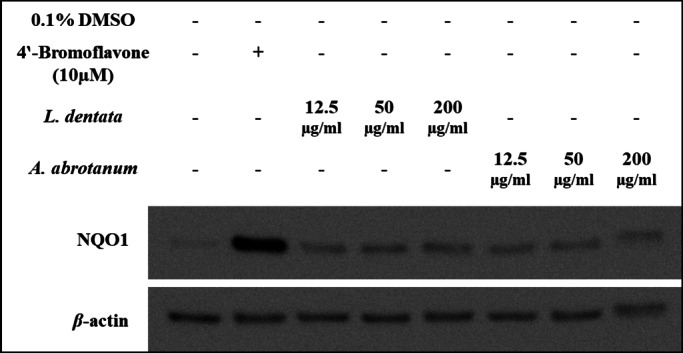
Concentration-dependent regulation of NQO1 protein expression by essential oils in Hepa1c1c7 cells. Indicated cells were cultured as monolayers and treated as mentioned in the Materials and Methods section. An uncropped image of all blots shown is provided in supplementary material as Figure S4.

**Fig. 6:**
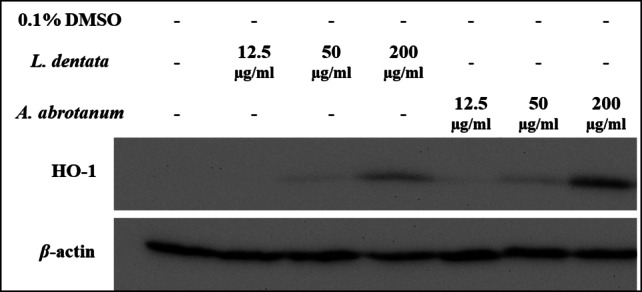
Concentration-dependent regulation of HO-1 protein expression by essential oils in Hepa1c1c7 cells. Indicated cells were cultured as monolayers and treated as mentioned in the Materials and Methods section. An uncropped image of all blots shown is provided in supplementary material as Figure S5.

An additional experiment was performed on the EOs of *A. Abrotanum* and *L. dentata* which showed a moderate ability to induce expression of NQO1, with increasing concentrations (12.5, 50 and 200 µg/ml). Also, a dose-response experiment was conducted to assay expression of HO-1 at 12.5, 50, 200 µg/ml. Different concentrations showed almost similar induction of the expression of chemo-preventive marker NQO1 protein as shown in Fig. 5. In addition, the induction of chemo-preventive marker HO-1 protein expression was increased at concentration-dependent, as shown by western blotting technique, and the maximum induction of HO-1 appeared at a concentration of 200 µg/ml, as illustrated in Fig. 6.

Essential oils have attracted considerable interest for their possible health advantages, especially concerning oxidative stress and inflammation. A significant area of investigation examines their impact on the Nrf2 signaling pathway, which is essential for regulating the body’s antioxidant response through the modulation of various cytoprotective genes, such as NQO1 and HO-1, with particular emphasis on their role in cancer chemoprevention.

## Discussion

The two major transcription factors; one responsible for cellular defense against oxidative stress and the other against inflammation—are Nrf2 and NF-κB, respectively. Although they are closely interrelated, these two major cellular stress-response systems often operate as functional antagonists. Their balance plays an important role in inflammation, oxidative stress, and cancer prevention. Loss of Nrf2 function may enhance NF-κB activity through increased cytokine production, while NF-κB modulates the transcription and activity of Nrf2 with both positive and negative influences on the target gene expression^64^.

In the present study, the EO of *A. abrotanum* exhibited strong anti-inflammatory potential against LPS-stimulated production of NO and expression of iNOS in RAW264.7 macrophages. Whereas the EO of *L. dentata* showed mediated anti-inflammatory effect. In contrast, the EO of *L. nobilis* exhibited low activity. These differences in biological activity can most likely be explained by differences in the chemical composition of the respective essential oils.

As shown in our previous publication^54^, the major constituents of *A. abrotanum* were artemisia ketone, *α*-eudesmol and *γ*-eudesmol. On the other hand, the most prominent compounds of *L. dentata* were eucalyptol, camphor and *α*-pinene.

To date, no studies have showed the anti-inflammatory properties of Artemisia ketone. On the other hand, *α*-eudesmol has shown promising anti-inflammatory activity through various molecular mechanisms. In an in silico study, Mondal et al.^65^ stated that *α*-eudesmol showed high binding affinity for crucial mediators of inflammation like interleukin-1β–converting enzyme, thus justifying its potential as a bioactive anti-inflammatory agent.

Eucalyptol is an established anti-inflammatory compound for which extensive evidence exists at the cellular, animal and clinical levels. It exerts anti-inflammatory effects by suppressing LPS–induced pro-inflammatory cytokines, including NF-κB, TNF-*α*, IL-1*β* and IL-6. It further modulates the extracellular signal-regulated kinase (ERK) pathway and diminishes oxidative stress by regulating redox-sensitive signalling and radical-scavenging activity^66^. Also, camphor has context-dependent and dose-sensitive immune-pharmacological properties, exhibiting both anti-inflammatory and potentially pro-inflammatory actions. Experimental work by Silva-Filho et al.^67^ showed that orally administered camphor inhibited leukocyte recruitment and reduced edema formation at a dosage of 100–200 mg/kg.

To the best of our knowledge, there are no previous studies that showed the anti-inflammatory activity of EO of *A. abrotanum*. However, the anti-inflammatory activity of different species of the genus *Аrtemisia* were previously published by other investigators. The inhibitory effect of the EO of *Аrtemisia fukudo* on the production of NO and PGE2 induced by LPS was due to suppression of iNOS and COX-2 expression^68^. It also inhibits NF-κB activation by preventing the nuclear translocation of p65 and p50 proteins. In addition, it is a powerful inhibitor of the production of NO, TNF-*α*, PGE2 and interleukins (IL-6 and IL-1*β*) in RAW264.7 cells induced by LPS and produces these effects through interaction at the transcription level. Furthermore, the anti-inflammatory effect of the EO of *Аrtemisia fukudo* was associated with the inactivation of MAPKs and NF-κB caused by blocking the phosphorylation of IkB-*α*, ERK, JNK and p38^68^. The EO of *Аrtemisia herba-alba* showed the efficacy to decrease the production of NO in RAW264.7 cells from 100 to 18.59 ± 1.26% at 1.25 µl/ml and BV-2 cells from 100 to 32.93 ± 1.51 at 1.25 µl/ml^69^. Zhu et al.^70^ demonstrated that the pre-treatment of RAW264.7 cells with the EO of *Аrtemisia annua* inhibit the production of IL-6, NO and TNF-*α* with inhibition rates 55.55, 47.84 and 26.57%, respectively. Also, Trinh et al.^71^ exhibited that the treatment of LPS-stimulate RAW264.7 cells with the EO of *Аrtemisia vulgaris* led to reduce NO generation by 9.48–58.43% at a gradual concentration from 12.5 to 100 µg/ml. In addition, the EO of *Аrtemisia vulgaris* decreased TNF-*α* by 11.6–55.6%.

The EO of *Lavandula viridis* exhibited significant inhibition of NO production by LPS in RAW264.7 cells with 96.15% inhibition at 0.64 µl/ml. In addition, pre-treatment of cells with the EO significantly suppressed iNOS and COX-2 protein expression by 97.40 and 53.9%, respectively, without any effect on the COX-1 level. It also caused significant inhibition of IκB-*α* phosphorylation and reduced the mRNA levels of iNOS and pro-inflammatory (IL-1*β* and IL-6)^72^. Wei et al.^73^ demonstrated that the EO of *Lavandula angustifolia* inhibited NO and ROS production in LPS-stimulated HaCaT (spontaneously immortalized human keratinocyte cells) and RAW264.7 cells, and suppressed gene expression of TNF-*α*, IL-6 and IL-1*β* at the protein and mRNA levels. 0.1% of EO reduced the production of ROS with 74.09 and 46.75%, respectively. It also had the suppression of iNOS protein expression at 0.01% of EO with inhibition rates of 49.49 and 32.49%, respectively. In addition, the EO at 0.001, 0.01 and 0.1% exhibited inhibition of p38 and JNK phosphorylation with inhibition rates ranging from 20.15 to 43.46%. In LPS-stimulated HaCaT cells, 0.1% of EO suppressed IκB*α* and p50 phosphorylation, with inhibition rates reaching to 65.10 and 60.44% but promoted p65 phosphorylation. On the other hand, in LPS-stimulated RAW264.7 cells, the EO at 0.001, 0.01 and 0.1% showed inhibitory effects against phosphorylation of IκB*α*, p65 and p50 with inhibition rates ranging from 10 to 50%.

The EO of *L. nobilis* significantly suppressed the progressive increase in inflammation in formaldehyde-induced paw edema in Wistar rats, in a dose-dependent manner up to 0.2 ml/kg body weight^74^. Al-Mijalli et al.^75^ demonstrated that the EO of *L. nobilis* have in vitro anti-inflammatory effect by inhibiting 5-Lipoxygenase (5-LOX) enzyme with IC_50_ value 48.31 ± 0.07 µg/ml, and it also significantly suppressed carrageenan-induced paw edema in Wistar rats with 70.59% inhibition at 50 mg/kg body weight. Jaradat et al.^45^ exhibited that the EO of *L. nobilis* showed an anti-inflammatory effect by inhibiting cyclooxygenase (COX) enzymes and exhibited 89.5 and 87.8% inhibition of COX-1 and COX-2 enzymes, respectively, at 40 µg/ml.

The EO of *C. citratus* exhibited a strong cytotoxic effect against RAW264.7 cells. This finding was agreement with a previous study by Sepúlveda-Arias et al.^76^ which showed the EO of *C. citratus* and citral exhibited a cytotoxic effect toward murine macrophage cells (RAW264.7). This toxicity prohibited further testing of the EO of *C. citratus* as anti-inflammatory activity should be at non-toxic concentrations. In addition, in our previous publication, we characterized the chemical composition and cytotoxic properties of the EOs under investigation, and demonstrated that the EO of *C. citratus* had a highly cytotoxic effect towards all evaluated cell lines; triple-negative breast cancer (MDA-MB-231), non-small lung adenocarcinoma (A549), colon adenocarcinoma (Caco2), hepatocellular carcinoma cells (HepG2) and doxorubicin-resistant hepatocellular carcinoma (HepG2/DOX)^54^.

We were unable to find any publications examining the cancer chemopreventive effects of essential oils distilled from the plant species under investigation. Other species within these genera, however, have been investigated. Also, to the best of our knowledge, there are no previous studies that have demonstrated the role of essential oil in cancer chemoprevention *via* the activated Nrf2 signaling pathway in murine hepatoma cells (Hepa1c1c7) model. While, it is evaluated through other models and techniques.

In a study by Li et al.^77^ Rosemary essential oil demonstrated the ability to alleviate oxidative stress by activating the Nrf2 signaling pathway, resulting in increased protein expression of NQO1 and other antioxidant enzymes such as HO-1.

The stimulation of the Nrf2-Keap1 signaling pathway is principally responsible for the induction of NQO1 by EO of *Artemisia vulgaris* and eucalyptol, which is a major compound. This route is essential for modulating the expression of several antioxidant enzymes, including NQO1. It also participates in the liver’s detoxification activities, so safeguarding against oxidative stress and hepatic injury^78^.

The regulation of the Nrf2 signaling pathway by essential oils offers a promising approach to augment cellular defense systems against oxidative stress. By augmenting the production of pivotal enzymes such as NQO1 and HO-1, these natural substances may provide therapeutic advantages in the management of illnesses linked to oxidative damage. Additional research is necessary to thoroughly clarify their mechanisms and their clinical uses.

## Conclusion

Anti-inflammatory activity was evaluated using RAW264.7 macrophages. The EO of *A. abrotanum* exhibited strong anti-inflammatory potential against LPS-induced NO on RAW264.7 cells. On the other hand, a cancer chemo-preventive influence was assessed in vitro utilizing Hepa1c1c7 murine carcinoma cells and showed that the EOs of *L. dentata* and *A. abrotanum* have moderate potency to induce protein expression of NQO1 and HO-1. These findings establish of *L. dentata* and *A. abrotanum* as promising chemopreventive and anti-inflammatory species for therapeutic development.

## Supplementary Information

Below is the link to the electronic supplementary material.

Supplementary Material 1

## Data Availability

All data generated or analysed during this study are included in this published article [and its supplementary information file].

## References

[1] Chopra, D. et al. Overview of inflammation. In *Inflammation Resolution and Chronic Diseases* (eds Tripathi, A., Dwivedi, A., Gupta, S. & Poojan, S.). 1–18. 10.1007/978-981-97-0157-5_1 (Springer Nature, 2024).

[2] Soares, C. L. R. et al. Biochemical aspects of the inflammatory process: A narrative review. *Biomed. Pharmacother*. **168**, 115764 (2023). 37897973 10.1016/j.biopha.2023.115764

[3] Piotrowski, I., Kulcenty, K. & Suchorska, W. Interplay between inflammation and cancer. *Rep. Pract. Oncol. Radiother*. **25**, 422–427 (2020). 32372882 10.1016/j.rpor.2020.04.004 PMC7191124

[4] Wen, Y. et al. Chronic inflammation, cancer development and immunotherapy. *Front. Pharmacol.* **13**, 1–16 (2022). 10.3389/fphar.2022.1040163 PMC9614255 36313280

[5] Multhoff, G., Molls, M. & Radons, J. Chronic inflammation in cancer development. *Front. Immunol.* **2**, 98 (2012). 22566887 10.3389/fimmu.2011.00098 PMC3342348

[6] Nishida, A. & Andoh, A. The role of inflammation in cancer: Mechanisms of tumor initiation, progression, and metastasis. *Cells* **14**, 488 (2025). 40214442 10.3390/cells14070488 PMC11987742

[7] Ahsan, H., Islam, S. U., Ahmed, M. B. & Lee, Y. S. Role of Nrf2, STAT3, and Src as molecular targets for cancer chemoprevention. *Pharmaceutics* **14**, 1775 (2022). 36145523 10.3390/pharmaceutics14091775 PMC9505731

[8] Siraj, M. A. et al. Cancer chemopreventive role of dietary terpenoids by modulating Keap1-Nrf2-ARE signaling system- A comprehensive update. *Appl. Sci.* **11**, 10806 (2021).

[9] Wu, X., Wei, J., Yi, Y., Gong, Q. & Gao, J. Activation of Nrf2 signaling: A key molecular mechanism of protection against cardiovascular diseases by natural products. *Front. Pharmacol.* **13**, 1057918 (2022). 36569290 10.3389/fphar.2022.1057918 PMC9772885

[10] Pouremamali, F., Pouremamali, A., Dadashpour, M., Soozangar, N. & Jeddi, F. An update of Nrf2 activators and inhibitors in cancer prevention/promotion. *Cell. Commun. Signal.* **20**, 100 (2022). 35773670 10.1186/s12964-022-00906-3 PMC9245222

[11] Chunarkar-Patil, P. et al. Anticancer drug discovery based on natural products: From computational approaches to clinical studies. *Biomedicines* **12**, 201 (2024). 38255306 10.3390/biomedicines12010201 PMC10813144

[12] Chaachouay, N. & Zidane, L. Plant-derived natural products: A source for drug discovery and development. *Drugs Drug Candidates*. **3**, 184–207 (2024).

[13] Nazir, I. & Gangoo, S. A. Pharmaceutical and therapeutic potentials of essential oils. In *Essential oils: Advances in Extractions and Biological Applications* (eds. Oliveira, M. S. De, Andrade, E. H. D. A. & Blumenberg, M.). Vol. 32. 125–138 (Books on Demand, 2022).

[14] Osaili, T. M. et al. A status review on health-promoting properties and global regulation of essential oils. *Molecules* **28**, 1809 (2023). 36838797 10.3390/molecules28041809 PMC9968027

[15] Chowdury, I. A. et al. Potential phytochemical, analgesic and anticancerous activities of *Cymbopogon citratus* leaf. *Am. J. Biomed. Res.* **3**, 66–70 (2015).

[16] Tavares, F. et al. *Cymbopogon citratus* industrial waste as a potential source of bioactive compounds. *J. Sci. Food Agric.* **95**, 2652–2659 (2015). 25389117 10.1002/jsfa.6999

[17] Almohawes, Z. N. & Alruhaimi, H. S. Effect of *Lavandula dentata* extract on ovalbumin-induced asthma in male Guinea pigs. *Brazilian J. Biol.* **80**, 87–96 (2020). 10.1590/1519-6984.191485 31017237

[18] Cubukcu, B. et al. In vitro antimalarial activity of crude extracts and compounds from *Artemisia abrotanum* L. *Phyther Res.* **4**, 203–204 (1990).

[19] Joghee, S. & Nagamani. Immunomodulatory activity of ethanolic extract of *Artemisia abrotanum*. *Int. J. Pharmacogn. Phytochem. Res.* **7**, 390–394 (2015).

[20] Elansary, H. O. et al. Polyphenol content and biological activities of *Ruta graveolens* L. and *Artemisia abrotanum* L. in Northern Saudi Arabia. *Processes* **8**, 531 (2020).

[21] Qnais, E. Y., Abdulla, F. A., Kaddumi, E. G. & Abdalla, S. S. Antidiarrheal activity of *Laurus nobilis* L. leaf extract in rats. *J. Med. Food*. **15**, 51–57 (2012). 22082096 10.1089/jmf.2011.1707

[22] Mohammed, R. R., Omer, A. K., Yener, Z., Uyar, A. & Ahmed, A. K. Biomedical effects of *Laurus nobilis* L. leaf extract on vital organs in streptozotocin-induced diabetic rats: Experimental research. *Ann. Med. Surg.* **61**, 188–197 (2021). 10.1016/j.amsu.2020.11.051 PMC7817776 33520200

[23] Ferreira, A., Proença, C., Serralheiro, M. L. M. & Araújo, M. E. M. The in vitro screening for acetylcholinesterase Inhibition and antioxidant activity of medicinal plants from Portugal. *J. Ethnopharmacol.* **108**, 31–37 (2006). 16737790 10.1016/j.jep.2006.04.010

[24] Kupeli, E., Orhan, I. & Yesilada, E. Evaluation of some plants used in Turkish folk medicine for their anti-inflammatory and antinociceptive activities. *Pharm. Biol.* **45**, 547–555 (2007).

[25] Rizwana, H., Al Kubaisi, N., Al-Meghailaith, N. N., Moubayed, N. M. & Albasher, G. Evaluation of chemical composition, antibacterial, antifungal, and cytotoxic activity of *Laurus nobilis* L grown in Saudi Arabia. *J. Pure Appl. Microbiol.* **13**, 2073–2085 (2019).

[26] Çöven, F. O. et al. Assessment of in-vitro cytotoxicity and in-ovo virucidal antiviral efficacy of various plant extracts and bioactive molecules. *Kafkas Univ. Vet. Fak. Derg.* **30**, 171–178 (2024).

[27] Umukoro, S., Ogboh, S. I., Omorogbe, O., Adekeye, A. L. A. & Olatunde, M. O. Evidence for the involvement of monoaminergic pathways in the antidepressant-like activity of *Cymbopogon citratus* in mice. *Drug Res. (Stuttg)*. **67**, 419–424 (2017). 28499312 10.1055/s-0043-106586

[28] Garba, H. A., Mohammed, A., Ibrahim, M. A. & Shuaibu, M. N. Effect of Lemongrass (*Cymbopogon citratus* Stapf) tea in a type 2 diabetes rat model. *Clin. Phytoscience*. **6**, 19 (2020).

[29] Salih, A. H., Salih, R. H. & Ahmed, H. Y. Bioactivity of *Cymbopogon citratus* aqueous extract against measles virus and some bacterial isolates. *Casp. J. Environ. Sci.* **20**, 585–592 (2022).

[30] Rahhal, B. et al. Multi-biological activity assessment and phytochemical characterization of an aqueous extract of the *Cymbopogon citratus* grown in Palestine. *BMC Complement. Med. Ther.* **24**, 27 (2024). 38195607 10.1186/s12906-024-04338-z PMC10775582

[31] Mothana, R. A. A., Gruenert, R., Bednarski, P. J. & Lindequist, U. Evaluation of the in vitro anticancer, antimicrobial and antioxidant activities of some Yemeni plants used in folk medicine. *Pharmazie* **64**, 260–268 (2009). 19435146

[32] Ali, M. A., Abul Farah, M., Al-Hemaid, F. M. & Abou-Tarboush, F. M. Vitro cytotoxicity screening of wild plant extracts from Saudi Arabia on human breast adenocarcinoma cells. *Genet. Mol. Res.* **13**, 3981–3990 (2014). 24938609 10.4238/2014.May.23.9

[33] Algieri, F. et al. Anti-inflammatory activity of hydroalcoholic extracts of *Lavandula dentata* L. and *Lavandula stoechas* L. *J. Ethnopharmacol.* **190**, 142–158 (2016). 27269390 10.1016/j.jep.2016.05.063

[34] Bashm, M. R., Rahmati, B. & Poorgholam, M. The effect of *Lavandula dentata* aerial parts hydroalcoholic extract on learning and memory in male streptozotocin-induced diabetic rat. *Daneshvar Med.* **27**, 1–8 (2019).

[35] Justus, B. et al. New insights into the mechanisms of French lavender essential oil on non-small-cell lung cancer cell growth. *Ind. Crops Prod.* **136**, 28–36 (2019).

[36] Benbrahim, C. et al. Chemical composition and biological activities of oregano and lavender essential oils. *Appl. Sci.* **11**, 5688 (2021).

[37] Tayeboon, G. S. et al. Effects of *Cymbopogon citratus* and ferula assa-foetida extracts on glutamate-induced neurotoxicity. *Vitr Cell. Dev. Biol. - Anim.* **49**, 706–715 (2013). 10.1007/s11626-013-9656-7 23949776

[38] Bharti, S. K., Kumar, A., Prakash, O., Krishnan, S. & Gupta, A. K. Essential oil of *Cymbopogon citratus* against diabetes: Validation by in vivo experiments and computational studies. *J. Bioanal Biomed.* **5**, 194–203 (2013).

[39] Madi, Y. F., Choucry, M. A., Meselhy, M. R. & El-Kashoury, E. S. A. Essential oil of *Cymbopogon citratus* cultivated in egypt: seasonal variation in chemical composition and anticholinesterase activity. *Nat. Prod. Res.* **35**, 4063–4067 (2021). 31960718 10.1080/14786419.2020.1713125

[40] Pallavi, P., Sahoo, P. P., Sen, S. K. & Raut, S. Comparative evaluation of anti-biofilm and anti-adherence potential of plant extracts against *Streptococcus mutans*: A therapeutic approach for oral health. *Microb. Pathog*. **188**, 106514 (2024). 38296118 10.1016/j.micpath.2023.106514

[41] Almahdawy, S. S., Said, A. M., Abbas, I. S. & Dawood, A. H. The evaluation of antimicrobial and cytotoxic activity of the essential oil extracted from the aerial parts of Southernwood herb (*Artemisia abrotanum* l.) that recently grown in Iraq. *Asian J. Pharm. Clin. Res.* **10**, 384–387 (2017).

[42] Sayyah, M., Valizadeh, J. & Kamalinejad, M. Anticonvulsant activity of the leaf essential oil of *Laurus nobilis* against pentylenetetrazole- and maximal electroshock-induced seizures. *Phytomedicine* **9**, 212–216 (2002). 12046861 10.1078/0944-7113-00113

[43] Yakoubi, R., Megateli, S., Sadok, H., Bensouici, T., Bağci, E. & C. & A synergistic interactions of Algerian essential oils of *Laurus nobilis* L., *Lavandula stoechas* L. and *Mentha pulegium* L. on anticholinesterase and antioxidant activities. *Biocatal. Agric. Biotechnol.* **31**, 101891 (2021).

[44] Ovidi, E. et al. *Laurus nobilis*, *Salvia sclarea* and *Salvia officinalis* essential oils and hydrolates: evaluation of liquid and vapor phase chemical composition and biological activities. *Plants* **10**, 707 (2021). 33917630 10.3390/plants10040707 PMC8067454

[45] Jaradat, N. et al. Chromatography analysis, in light of vitro antioxidant, antidiabetic, antiobesity, anti-inflammatory, antimicrobial, anticancer, and three-dimensional cancer spheroids’ formation blocking activities of *Laurus nobilis* aromatic oil from Palest. *Chem. Biol. Technol. Agric.* **10**, 25 (2023).

[46] El Abdali, Y. et al. *Lavandula dentata* L.: Phytochemical analysis, antioxidant, antifungal and insecticidal activities of its essential oil. *Plants* **11**, 311 (2022). 10.3390/plants11030311 PMC8840530 35161292

[47] Ali, M. et al. (ed, K.) In vitro characterization of the essential oil extracted from Lavandula dentata and its application in the field of biotherapy. *Pakistan J. Biol. Sci.* **26** 300–310 (2023). 10.3923/pjbs.2023.300.310 37902044

[48] Moreira, F. V., Bastos, J. F. A., Blank, A. F., Alves, P. B. & Santos, M. R. V. Chemical composition and cardiovascular effects induced by the essential oil of *Cymbopogon citratus* DC. Stapf, Poaceae, in rats. *Brazilian J. Pharmacogn*. **20**, 904–909 (2010).

[49] de Costa, C. A. R. The GABAergic system contributes to the anxiolytic-like effect of essential oil from *Cymbopogon citratus* (lemongrass). *J. Ethnopharmacol.* **137**, 828–836 (2011). 21767622 10.1016/j.jep.2011.07.003

[50] Costa, C. A. R. A. et al. Cholesterol reduction and lack of genotoxic or toxic effects in mice after repeated 21-day oral intake of lemongrass (*Cymbopogon citratus*) essential oil. *Food Chem. Toxicol.* **49**, 2268–2272 (2011). 21693164 10.1016/j.fct.2011.06.025

[51] Mukhtar, M. H. et al. *Cymbopogon citratus* and citral overcome doxorubicin resistance in cancer cells via modulating the drug’s metabolism, toxicity, and multidrug transporters. *Molecules* **28**, 3415 (2023). 37110649 10.3390/molecules28083415 PMC10143904

[52] Boukhatem, M. N., Ferhat, M. A., Kameli, A., Saidi, F. & Kebir, H. T. Lemon grass (*Cymbopogon citratus*) essential oil as a potent anti-inflammatory and antifungal drugs. *Libyan J. Med.* **9**, 25431 (2014). 25242268 10.3402/ljm.v9.25431 PMC4170112

[53] Silva, M. R. et al. Comparative anticonvulsant activities of the essential oils (EOs) from *Cymbopogon winterianus* Jowitt and *Cymbopogon citratus* (DC) Stapf. In mice. *Naunyn Schmiedebergs Arch. Pharmacol.* **381**, 415–426 (2010). 20237771 10.1007/s00210-010-0494-9

[54] Ali, M. I. et al. Chemical composition, antioxidant, and cytotoxic potential of essential oils of some cultivated plants in Egypt. *Egypt. J. Chem.* **68**, 555–571 (2025).

[55] Guenther, E. *The Essential Oils*. Vol. 1. 427 (D.Van Nostrand Company, Inc., 1948).

[56] Hamed, A. R. et al. Salvimulticanol from salvia multicaulis suppresses LPS-induced inflammation in RAW264.7 macrophages: In vitro and in silico studies. *3 Biotech.* **14**, 144 (2024). 38706927 10.1007/s13205-024-03987-8 PMC11065832

[57] Aboshouk, D. R. et al. Curcumin mimics of potential chemoprevention with NQO1 induction properties. *Sci. Rep.* **15**, 2332 (2025). 39824830 10.1038/s41598-025-85588-w PMC11748699

[58] Dirsch, V. M., Stuppner, H. & Vollmar, A. M. The Griess assay: Suitable for a bio-guided fractionation of anti- inflammatory plant extracts. *Planta Med.* **64**, 423–426 (1998). 9690344 10.1055/s-2006-957473

[59] Hamed, A. R., Ali, M. I., El-Shiekh, R. A., Abdel-Sattar, E. & Fayek, N. M. Potential chemo preventive and anti-inflammatory constituents from *Centaurea pumilio*: Bio-guided isolation. *Nat. Prod. Res.*10.1080/14786419.2025.2515273 (2025). 40522348 10.1080/14786419.2025.2515273

[60] Jang, H. Y. & Lee, S. Heme oxygenase 1-mediated anti-inflammatory effect of extract from the aerial part of *Heracleum moellendorffii* hance. *Foods* **12**, 3309 (2023). 37685243 10.3390/foods12173309 PMC10486398

[61] Bode, J. G., Ehlting, C. & Häussinger, D. The macrophage response towards LPS and its control through the p38 MAPK - STAT3 axis. *Cell. Signal.* **24**, 1185–1194 (2012). 22330073 10.1016/j.cellsig.2012.01.018

[62] Gyorfy, Z., Duda, E. & Vizler, C. Interactions between LPS moieties and macrophage pattern recognition receptors. *Vet. Immunol. Immunopathol.* **152**, 28–36 (2013). 23084343 10.1016/j.vetimm.2012.09.020

[63] Tossetta, G. et al. The role of NQO1 in ovarian cancer. *Int. J. Mol. Sci.* **24**, 7839 (2023). 37175546 10.3390/ijms24097839 PMC10178676

[64] Wardyn, J. D., Ponsford, A. H. & Sanderson, C. M. Dissecting molecular cross-talk between Nrf2 and NF-κB response pathways. *Biochem. Soc. Trans.* **43**, 621–626 (2015). 26551702 10.1042/BST20150014 PMC4613495

[65] Mondal, M. et al. Analgesic and anti-inflammatory potential of essential oil of eucalyptus camaldulensis leaf: In vivo and in Silico studies. *Nat. Prod. Commun.* **16**, 1–16 (2021).

[66] Seol, G. H. & Kim, K. Y. Eucalyptol and its role in chronic diseases. In *Advances in Experimental Medicine and Biology: Drug Discovery from Mother Nature* (eds. Gupta, S. C., Prasad, S. & Aggarwal, B. B.). Vol. 929. 389–398 (Springer,, 2016). 10.1007/978-3-319-41342-6_18 10.1007/978-3-319-41342-6_18 27771935

[67] Silva-filho, S. E. et al. Effect of camphor on the behavior of leukocytes in vitro and in vivo in acute inflammatory response. *Trop. J. Pharm. Res.* **13**, 2031–2037 (2014).

[68] Yoon, W. J. et al. Artemisia fukudo essential oil attenuates LPS-induced inflammation by suppressing NF-κB and MAPK activation in RAW 264.7 macrophages. *Food Chem. Toxicol.* **48**, 1222–1229 (2010). 10.1016/j.fct.2010.02.014 20156520

[69] Abu-Darwish, M. S. et al. Artemisia herba-alba essential oil from Buseirah (South Jordan): Chemical characterization and assessment of safe antifungal and anti-inflammatory doses. *J. Ethnopharmacol.* **174**, 153–160 (2015). 10.1016/j.jep.2015.08.005 26277492

[70] Zhu, C. et al. Phytochemical profile and anti-inflammatory effect of *Artemisia annua* L. essential oil. *All Life*. **16**, 2288529 (2023).

[71] Trinh, P. T. N. et al. Antioxidant, anti-inflammatory, and anti-bacterial activities of *Artemisia vulgaris* L. essential oil in Vietnam. *Nat. Prod. Commun.* **19** (2024).

[72] Zuzarte, M. et al. *Lavandula viridis* L´Hér. essential oil inhibits the inflammatory response in macrophages through blockade of NF-KB signaling cascade. *Front. Pharmacol.* **12**, 695911 (2022). 35145398 10.3389/fphar.2021.695911 PMC8821966

[73] Wei, M. et al. In vitro and in silico analysis of ‘Taikong blue’ lavender essential oil in LPS–induced HaCaT cells and RAW264.7 murine macrophages. *BMC Complement. Med. Ther.* **22**, 324 (2022). 10.1186/s12906-022-03800-0 PMC9727978 36474235

[74] Sayyah, M., Saroukhani, G., Peirovi, A. & Kamalinejad, M. Analgesic and anti-inflammatory activity of the leaf essential oil of *Laurus nobilis* Linn. *Phyther Res.* **17**, 733–736 (2003). 10.1002/ptr.1197 12916069

[75] Al-Mijalli, S. H. et al. Chemical composition, antioxidant, anti-diabetic, anti-acetylcholinesterase, anti-inflammatory, and antimicrobial properties of *Arbutus unedo* L. and *Laurus nobilis* L. essential oils. *Life* **12**, 1876 (2022). 36431011 10.3390/life12111876 PMC9695135

[76] Sepúlveda-Arias, J. C., Veloza, L. A., Escobar, L. M., Orozco, L. M. & Lopera, I. A. Anti-inflammatory effects of the main constituents and epoxides derived from the essential oils obtained from *Tagetes lucida*, *Cymbopogon citratus*, *Lippia alba* and *Eucalyptus citriodora*. *J. Essent. Oil Res.* **25**, 186–193 (2013).

[77] Li, Y., Huang, L., Xu, Y., Cheng, B. & Zhao, M. Optimization of enzyme-assisted extraction of rosemary essential oil using response surface methodology and its antioxidant activity by activating Nrf2 signaling pathway. *Molecules* **29**, 3382 (2024). 39064960 10.3390/molecules29143382 PMC11279388

[78] Jiang, Z. et al. The essential oils and eucalyptol from *Artemisia vulgaris* L. prevent acetaminophen-induced liver injury by activating Nrf2-Keap1 and enhancing APAP clearance through non-toxic metabolic pathway. *Front. Pharmacol.* **10**, 782 (2019). 31404264 10.3389/fphar.2019.00782 PMC6669816

