# Supplementary material for: Anti-inflammatory and cancer chemopreventive potential of essential oils from some cultivated plants in Egypt
Source: Sci Rep. 2026 Jan 29;16:4389. doi: 10.1038/s41598-026-35195-0 (PMC12864766; doi:10.1038/s41598-026-35195-0)
Supplement: Supplementary file 1 — Supplementary Material 1 [file 41598_2026_35195_MOESM1_ESM.docx]

**Supplementary material**

**Anti-inflammatory and cancer chemopreventive potential of essential oils from some cultivated plants in Egypt**

**Mohammed I. Ali^1^, Ahmed R. Hamed^2^, Emad M. Hassan^1^, Faten M. Abou elella^3^, Sayed A. El-Toumy^4^ and Ahmed M. Aboul-Enein**

^1^Medicinal and Aromatic Plants Research Department, National Research Centre, Dokki, Giza, 12622, Egypt

^2^Chemistry of Medicinal Plants Department, National Research Centre, Dokki, Giza, 12622, Egypt

^3^Biochemistry Department, Faculty of Agriculture, Cairo University, Giza, 12613, Egypt

^4^Chemistry of Tannins Department, National Research Centre, Dokki, Giza, 12622, Egypt

***Abstract***

***This study*** ***aimed to*** investigate the biological activity of essential oils (EOs) of *Artemisia abrotanum*, *Lavandula dentata*, *Cymbopogon citratus* and *Laurus nobilis* as anti-inflammatory and cancer chemopreventive activities. ***Methods:*** the anti-inflammatory activity was evaluated using lipopolysaccharides-induced nitric oxide (NO) inhibition on murine macrophage cells (RAW264.7) and cancer chemo-preventive influence was assessed *in vitro* utilizing Hepa1c1c7 murine carcinoma cells. ***Results:*** the EO of *A. abrotanum* has the most potential activity toward inhibition of NO release, recording 96.6±0.1%, as estimated by Greiss assay. Followed by EOs of *L. dentata* and *L. nobilis* with 63.6±0.11 and 37.0±0.23%, respectively. At the protein expression level, western blotting technique was used to evaluate the expression of iNOS. The EO of *A. abrotanum* at 100 µg/ml exhibited a very high impact on inhibiting iNOS expression, followed by EOs of *L. dentata* and *L. nobilis*. On the other hand, pre-screening concentration (100 µg/ml) revealed that the EOs of *L. dentata* and *A. abrotanum* have moderate potency to induce expression of chemo-preventive marker NQO1. ***Conclusion:*** The results revealed that the EO of *A. abrotanum* had strong anti-inflammatory activity. While the EOs of *L. dentata* and *A. abrotanum* have moderate potency to induce cancer chemoprevention.

**Keywords:** Essential oils; Medicinal plants; Anti-inflammatory and Cancer chemopreventive.

| Figure | Page |
| --- | --- |
| **Fig. S1.** Uncropped western blotting of iNOS inhibition by essential oils at single concentration (100µg/ml) as shown on Figure 2 in the manuscript. Image background was adjusted to show the blot membrane strips and developed bands at the same time. Blot was cutting to 3 strips; upper strip for iNOS (A), empty strip (B) and bottom strip for β-actin (C). (I) the membrane strips digital image with normal light, (II) the intensity-tuned image to reveal the membrane strip shadow, (III) the auto-tuned and uncropped image and (IV) the main manuscript cropped blot image for direct comparison. Red squares refer to the target samples in the present study and red arrows refer to target protein bands. RAW264.7 cells were cultured as monolayers and treated as mentioned at the Materials and Methods sections. | 3 |
| **Fig. S2.** Uncropped western blotting of iNOS inhibition by essential oil of *A. abrotanum* at different concentration as shown on Figure 3 in the manuscript. Image background was adjusted to show the blot membrane strips and developed bands at the same time. Blot was cutting to 3 strips; upper strip for iNOS (A), middle strip of the blot irrelevant to study targets (B) and bottom strip for β-actin (C). (I) the membrane strips digital image with normal light, (II) the intensity-tuned image to reveal the membrane strip shadow, (III) the auto-tuned and uncropped image and (IV) the main manuscript cropped blot image for direct comparison. Red squares refer to the target samples in the present study and red arrows refer to target protein bands. RAW264.7 cells were cultured as monolayers and treated as mentioned at the Materials and Methods sections. | 4 |
| **Fig. S3.** Uncropped western blotting of NOQ1 induction by essential oils at single concentration (100µg/ml) as shown on Figure 4 in the manuscript. Image background was adjusted to show the blot membrane strips and developed bands at the same time. Blot was cutting to 2 strips; upper strip for NQO1 (A) and bottom strip for β-actin (B). (I) the intensity-tuned image to reveal the membrane strip shadow, (II) the auto-tuned and uncropped image and (III) the main manuscript cropped blot image for direct comparison. Red squares refer to the target samples in the present study and red arrows refer to target protein bands. Hepa1c1c7 cells were cultured as monolayers and treated as mentioned at the Materials and Methods sections. | 5 |
| **Fig. S4.** Uncropped western blotting of NQO1 induction by active essential oils at different concentration as shown on Figure 5 in the manuscript. Image background was adjusted to show the blot membrane strips and developed bands at the same time. Blot was cutting to 2 strips; upper strip for NQO1 (A) and bottom strip for β-actin (B). (I) the membrane strips digital image with normal light, (II) the intensity-tuned image to reveal the membrane strip shadow, (III) the auto-tuned and uncropped image and (IV) the main manuscript cropped blot image for direct comparison. Red squares refer to the target samples in the present study and red arrows refer to target protein bands. Hepa1c1c7 cells were cultured as monolayers and treated as mentioned at the Materials and Methods sections. | 6 |
| **Fig. S5.** Uncropped western blotting of HO-1 induction by active essential oils at different concentration as shown on Figure 6 in the manuscript. Image background was adjusted to show the blot membrane strips and developed bands at the same time. Blot was cutting to 2 strips; upper strip for HO-1 (A) and bottom strip for β-actin (B). (I) the intensity-tuned image to reveal the membrane strip shadow, (II) the auto-tuned and uncropped image and (III) the main manuscript cropped blot image for direct comparison. Red squares refer to the target samples in the present study and red arrows refer to target protein bands. Hepa1c1c7 cells were cultured as monolayers and treated as mentioned at the Materials and Methods sections. | 7 |


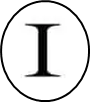

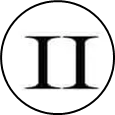

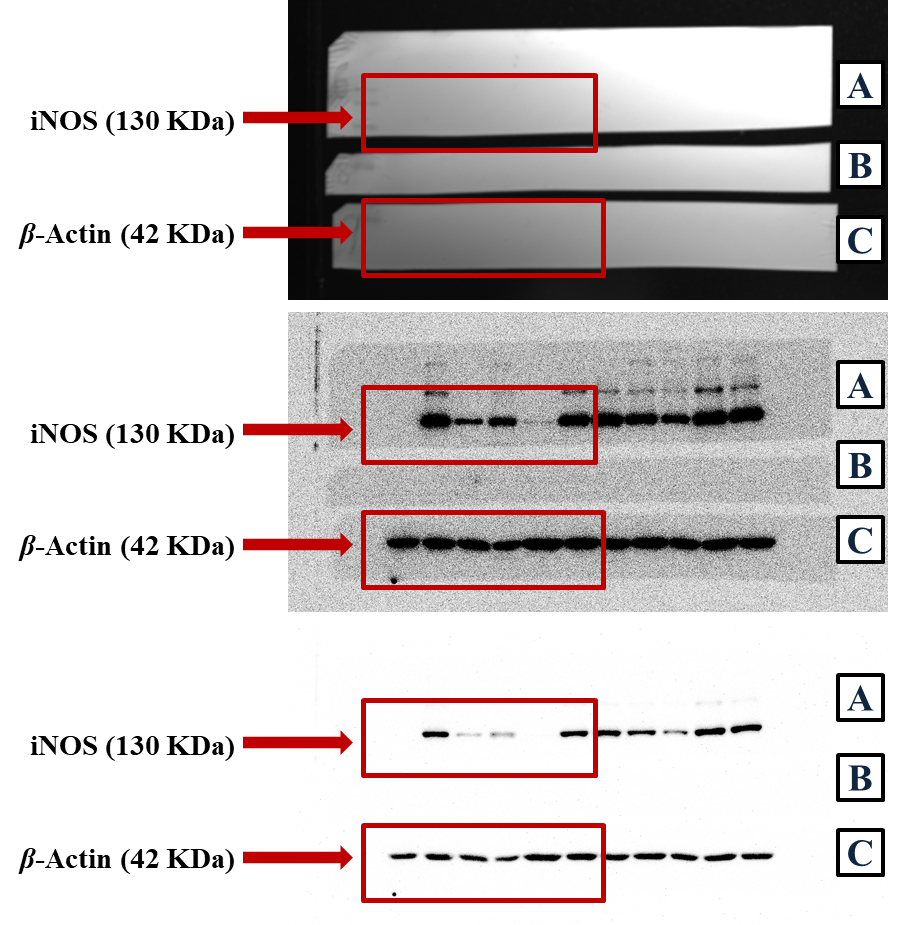

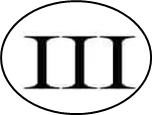

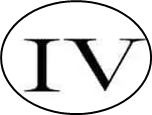

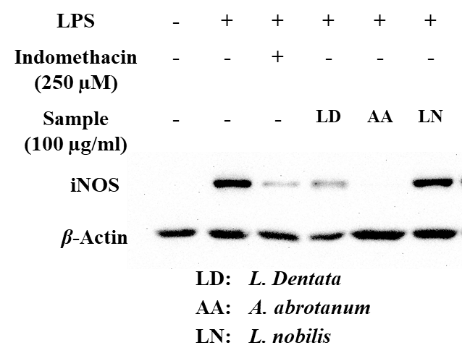


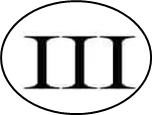
**Fig. S1.** Uncropped western blotting of iNOS inhibition by essential oils at single concentration (100µg/ml) as shown on Figure 2 in the manuscript. Image background was adjusted to show the blot membrane strips and developed bands at the same time. Blot was cutting to 3 strips; upper strip for iNOS (A), empty strip (B) and bottom strip for *β*-actin (C). (I) the membrane strips digital image with normal light, (II) the intensity-tuned image to reveal the membrane strip shadow, (III) the auto-tuned and uncropped image and (IV) the main manuscript cropped blot image for direct comparison. Red squares refer to the target samples in the present study and red arrows refer to target protein bands. RAW264.7 cells were cultured as monolayers and treated as mentioned at the Materials and Methods sections.


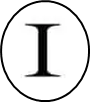

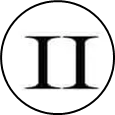

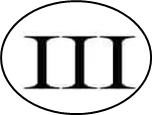

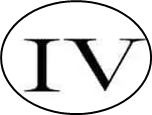

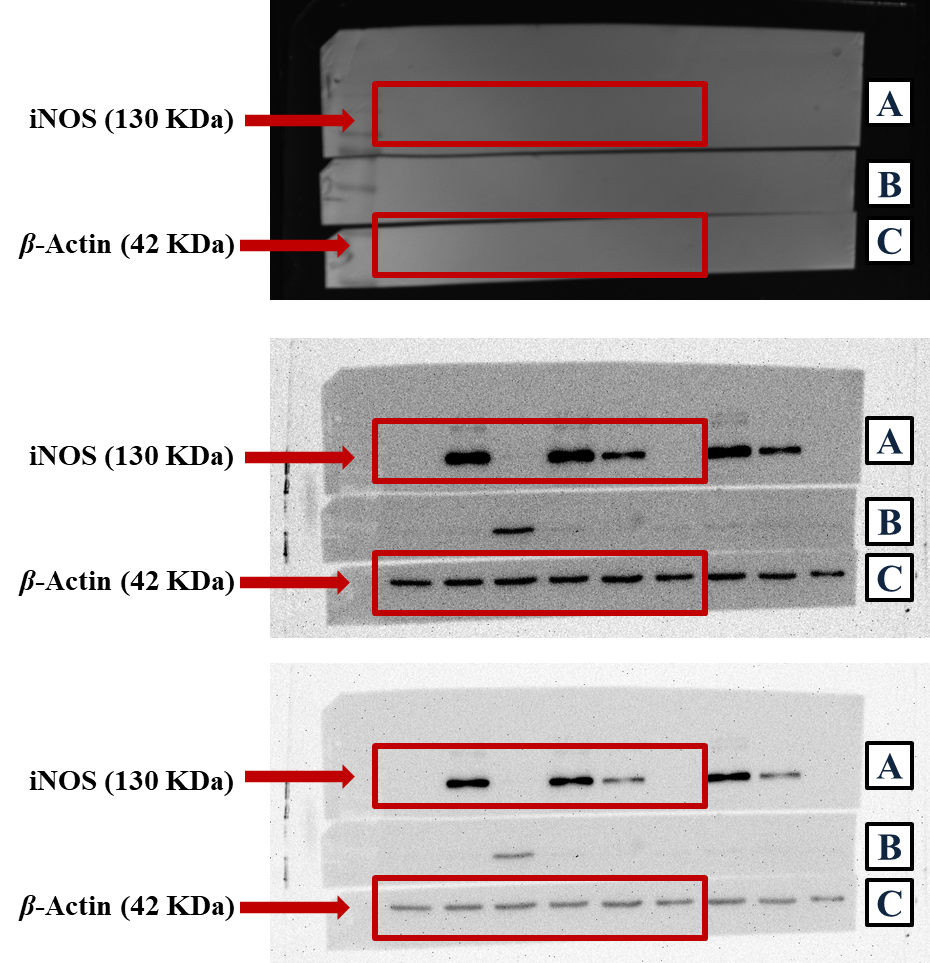

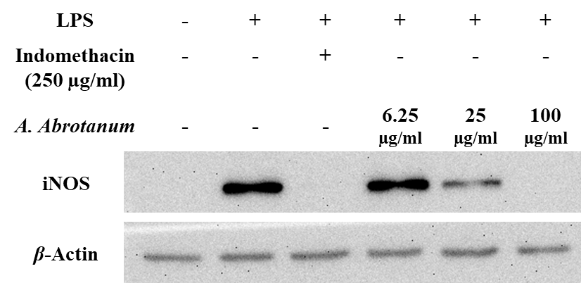


**Fig. S2.** Uncropped western blotting of iNOS inhibition by essential oil of *A. abrotanum* at different concentration as shown on Figure 3 in the manuscript. Image background was adjusted to show the blot membrane strips and developed bands at the same time. Blot was cutting to 3 strips; upper strip for iNOS (A), middle strip of the blot irrelevant to study targets (B) and bottom strip for β-actin (C). (I) the membrane strips digital image with normal light, (II) the intensity-tuned image to reveal the membrane strip shadow, (III) the auto-tuned and uncropped image and (IV) the main manuscript cropped blot image for direct comparison. Red squares refer to the target samples in the present study and red arrows refer to target protein bands. RAW264.7 cells were cultured as monolayers and treated as mentioned at the Materials and Methods sections.


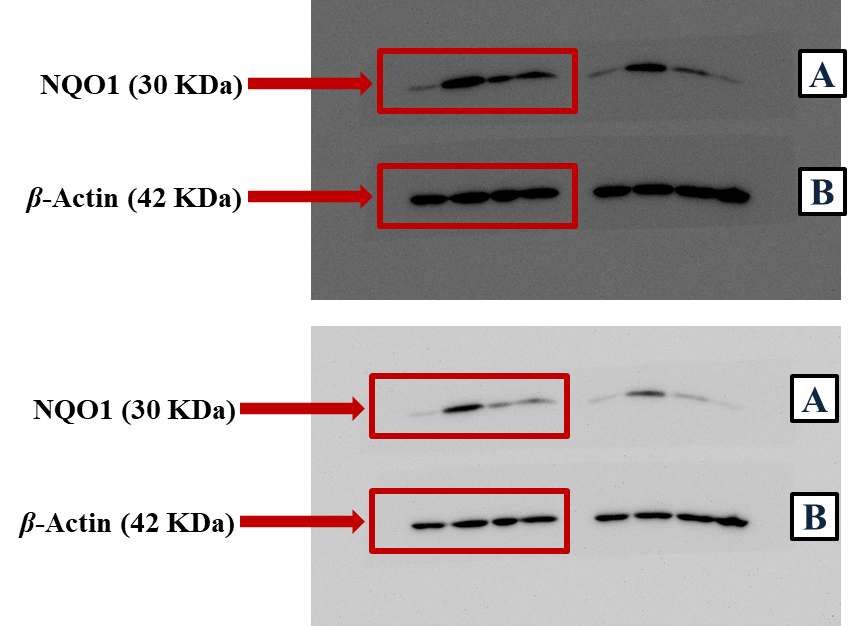

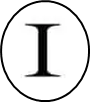

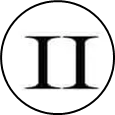

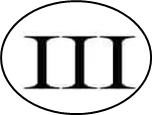

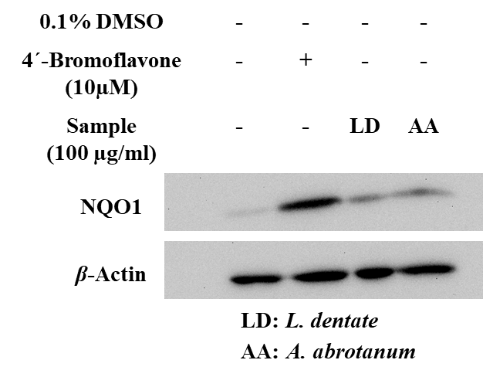


**Fig. S3.** Uncropped western blotting of NOQ1 induction by essential oils at single concentration (100µg/ml) as shown on Figure 4 in the manuscript. Image background was adjusted to show the blot membrane strips and developed bands at the same time. Blot was cutting to 2 strips; upper strip for NQO1 (A) and bottom strip for β-actin (B). (I) the intensity-tuned image to reveal the membrane strip shadow, (II) the auto-tuned and uncropped image and (III) the main manuscript cropped blot image for direct comparison. Red squares refer to the target samples in the present study and red arrows refer to target protein bands. Hepa1c1c7 cells were cultured as monolayers and treated as mentioned at the Materials and Methods sections.


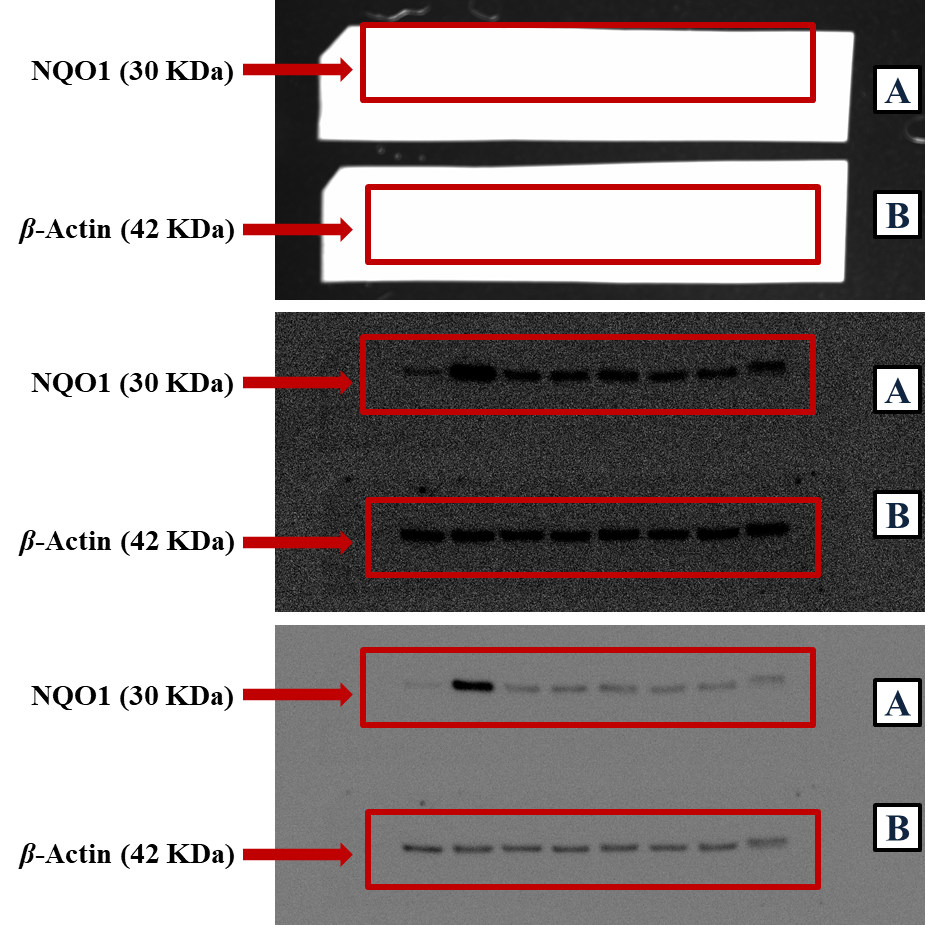

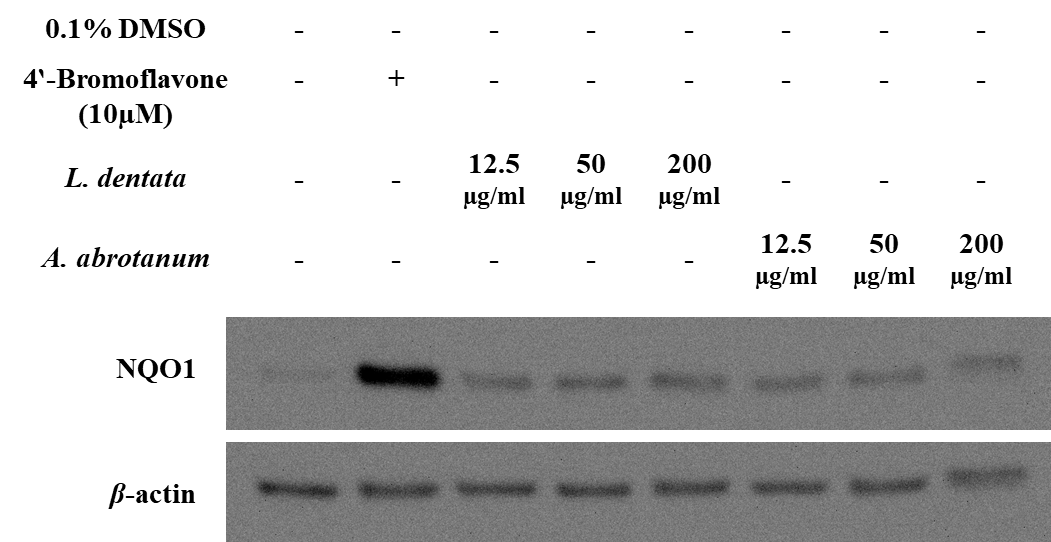

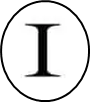

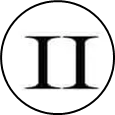

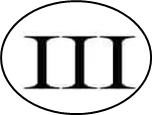

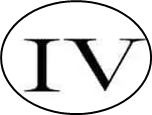


**Fig. S4.** Uncropped western blotting of NQO1 induction by active essential oils at different concentration as shown on Figure 5 in the manuscript. Image background was adjusted to show the blot membrane strips and developed bands at the same time. Blot was cutting to 2 strips; upper strip for NQO1 (A) and bottom strip for β-actin (B). (I) the membrane strips digital image with normal light, (II) the intensity-tuned image to reveal the membrane strip shadow, (III) the auto-tuned and uncropped image and (IV) the main manuscript cropped blot image for direct comparison. Red squares refer to the target samples in the present study and red arrows refer to target protein bands. Hepa1c1c7 cells were cultured as monolayers and treated as mentioned at the Materials and Methods sections.


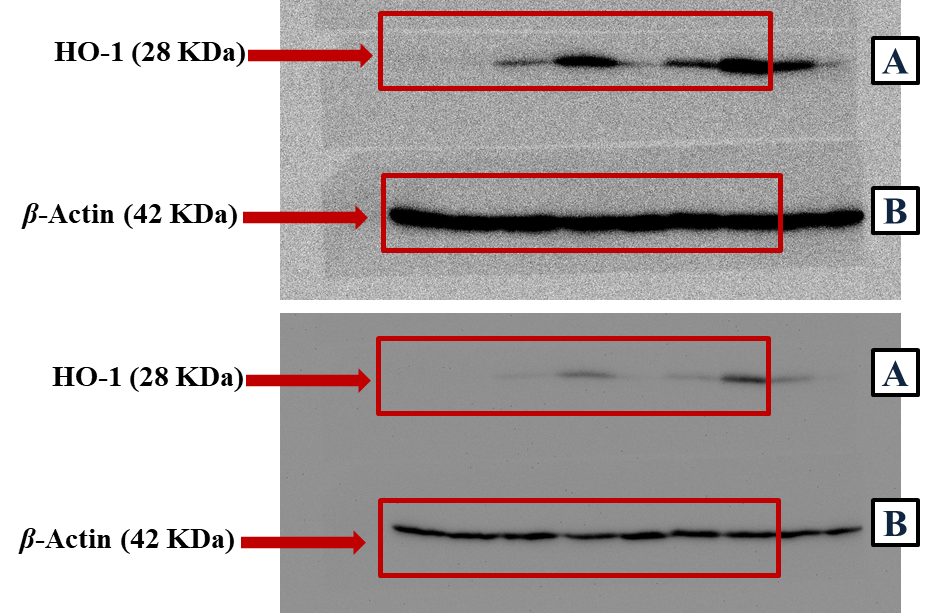

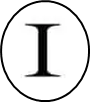

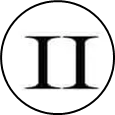

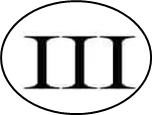

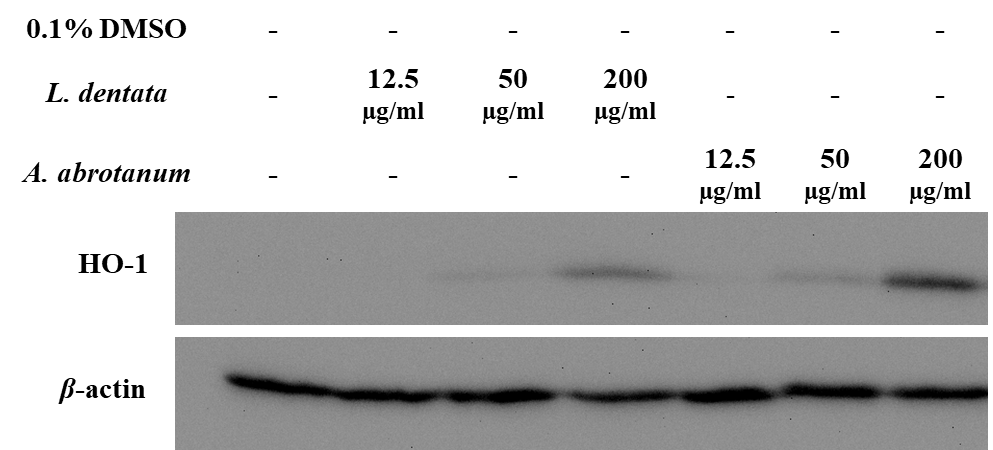


**Fig. S5.** Uncropped western blotting of HO-1 induction by active essential oils at different concentration as shown on Figure 6 in the manuscript. Image background was adjusted to show the blot membrane strips and developed bands at the same time. Blot was cutting to 2 strips; upper strip for HO-1 (A) and bottom strip for *β*-actin (B). (I) the intensity-tuned image to reveal the membrane strip shadow, (II) the auto-tuned and uncropped image and (III) the main manuscript cropped blot image for direct comparison. Red squares refer to the target samples in the present study and red arrows refer to target protein bands. Hepa1c1c7 cells were cultured as monolayers and treated as mentioned at the Materials and Methods sections.
